# Normocalcemic Hyperparathyroidism: A Heterogeneous Disorder Often Misdiagnosed?

**DOI:** 10.1002/jbm4.10391

**Published:** 2020-07-24

**Authors:** Guido Zavatta, Bart L Clarke

**Affiliations:** ^1^ Department of Medical and Surgical Sciences, University of Bologna Italy; ^2^ Division of Endocrinology, Diabetes, Metabolism and Nutrition, Mayo Clinic Rochester Minnesota USA

**Keywords:** HYPERCALCEMIA, HYPERCALCIURIA, NEPHROLITHIASIS, NORMOCALCEMIC HYPERPARATHYROIDISM, OSTEOPOROSIS, PRIMARY HYPERPARATHYROIDISM, SECONDARY HYPERPARATHYROIDISM

## Abstract

Normocalcemic primary hyperparathyroidism (NHPT) was first described over 10 years ago, but uncertainties still remain about its definition, prevalence, and rates of complications. As a result, consensus management guidelines for this condition have not yet been published. Several hypotheses have been proposed for the pathophysiology of NHPT, but it may be a heterogeneous disorder with multiple causes, rather than a single etiology that explains this biochemical phenotype. A common clinical concern is whether NHPT should be treated surgically when complications are already present at first recognition of the disorder, rather than following patients clinically over time. The literature on NHPT is based mostly on larger studies of population‐based cohorts and smaller studies from referral centers. Lack of rigorous diagnostic criteria and selection bias inherent in populations seen at tertiary referral centers may explain the heterogeneity of reported rates of bone and renal complications in relation to consistently mild laboratory alterations. Unresolved questions remain about the significance of NHPT when it is diagnosed biochemically without evident bone or kidney complications. Moreover, its natural history remains to be elucidated because a proportion of what is classified as NHPT may revert to normal spontaneously, thus revealing previously unrecognized secondary hyperparathyroidism. These issues indicate that caution should be used in recommending surgery for NHPT. This review will focus on recent issues regarding the pathophysiology, evaluation, and management of NHPT. © 2020 The Authors. *JBMR Plus* published by Wiley Periodicals LLC on behalf of American Society for Bone and Mineral Research.

## Introduction

Normocalcemic primary hyperparathyroidism (NHPT) is the newest phenotype of pathologic parathyroid overactivity, initially formally recognized and defined as a distinct entity in 2008 at the Third International Workshop on the Management of Asymptomatic Primary Hyperparathyroidism.^(^ ^1^ ^)^ This marked the recognition of the third subtype presentation of primary hyperparathyroidism (PHPT),^(^ ^2^ ^)^ which was initially described with classical manifestations and symptoms in the early 1900s, then as the more frequently recognized asymptomatic form with the advent of automated calcium (Ca) analyzers in the 1970s. The asymptomatic subtype is currently the most common phenotype of PHPT seen in Europe and North America.^(^ ^3^ ^)^

A significant number of studies on NHPT have been published since 2008 with the aim of further characterizing this disorder. In two previous reviews, Cusano and colleagues extensively addressed the etiology and management of this disease.^(^ ^2^, ^4^ ^)^ Over 10 years have passed and there is not yet a consensus for recommending either conservative medical management or surgical treatment of NHPT.

Rates of bone and renal complications described in NHPT vary considerably according to the type of population studied, thereby making it difficult to draw firm conclusions as to the pathogenetic role of excessive PTH in this setting. Moreover, there are no consistent data about the natural history of the disorder or the potential worsening of pre‐existing complications during long‐term follow‐up. Consequently, the benefits of aggressive therapeutic approaches in NHPT remain to be demonstrated.

The heterogeneous results in the literature regarding NHPT and its complications may be explained by two factors: the widespread failure of many studies to include subjects that rigorously fulfilled the full diagnostic criteria for NHPT, and the persistent uncertainty regarding the pathophysiology underlying NHPT. This review will detail the common issues related to NHPT and present recent advances in this field when the condition is diagnosed prior to initial parathyroidectomy. In addition, it will focus on the definition and diagnosis of the disorder, its prevalence, recognized complications, and the few outcomes reported to date based on different therapeutic approaches.

## Definition and Diagnosis

Normocalcemic hyperparathyroidism was first acknowledged to be part of the diagnostic spectrum of PHPT during the Third International Workshop on the Management of Asymptomatic Primary Hyperparathyroidism in 2008,^(^ ^1^ ^)^ and further defined and characterized at the Fourth International Workshop that followed in in 2014.^(^ ^5^ ^)^ NHPT is characterized by persistently increased serum PTH levels in the setting of normal albumin‐adjusted and ionized serum Ca, after secondary causes of PTH elevation have been excluded. The consensus statement from the Fourth International Workshop indicated that these laboratory findings should be confirmed on at least two occasions over a time frame of at least 3 to 6 months.

Based on this definition, NHPT is necessarily a diagnosis of exclusion. Because multiple causes of increased PTH secretion have been identified, and because the disorder does not generally warrant immediate surgical action, it is advisable to thoroughly rule out all possible factors contributing to secondary hyperparathyroidism. The following medical conditions or comorbidities must be ruled out to arrive at a correct diagnosis of NHPT: vitamin D deficiency (<20 to 30 ng/mL); insufficient Ca intake or Ca malabsorption; chronic kidney disease (eGFR <60 mL/min); medications including loop diuretics, thiazide diuretics, lithium, bisphosphonates, or denosumab; and idiopathic hypercalciuria with or without nephrolithiasis.

This rigorous definition should distinguish NHPT from the many other causes of hyperparathyroidism that after appropriate evaluation and management turn out to be either forms of classical PHPT or secondary hyperparathyroidism. Vitamin D deficiency may lower serum Ca or increase PTH levels in PHPT. By increasing vitamin D levels into an optimal range, Ca levels may increase because of improved intestinal Ca absorption, thereby changing the presumed diagnosis of NHPT to PHPT. There is uncertainty over the minimum threshold of vitamin D necessary for skeletal health.^(^ ^6^ ^)^ Some guidelines favor ≥20 ng/mL, whereas others recommend ≥30 ng/mL.^(^ ^7^, ^8^ ^)^ However, these threshold levels may not be sufficient to normalize PTH in all patients.^(^ ^9^ ^)^ To be confident of a diagnosis of NHPT, it is necessary that serum 25‐hydroxyvitamin D [25(OH)D] be within the optimal range defined by the laboratory, with a minimum level of 30 ng/mL. Some patients might require higher levels within the optimal range to normalize their PTH secretion, suggesting that a range of optimal vitamin D values rather than a single threshold might be appropriate for an accurate diagnosis of NHPT. Accurate vitamin D assays are also essential for correct interpretation of the laboratory data.^(^ ^10^ ^)^

Moreover, proper counseling of patients regarding adequate dietary Ca intake may result in improved Ca intake and absorption, with retrospective recognition of a previous secondary form of hyperparathyroidism rather than PHPT. Adequate investigation and evaluation of the differential diagnosis of hypercalciuria must also be conducted. Primary renal Ca loss over many years may secondarily increase PTH levels, eventually leading to chronic secondary hyperparathyroidism,^(^ ^11^ ^)^ which ultimately leads to parathyroid autonomy with resultant multigland parathyroid hyperplasia. Therefore, before confirming a diagnosis of NHPT when urinary Ca is significantly increased (>350 mg/day in females, or > 400 mg/day in males, or >4 mg/kg/day), a short‐term trial of a low‐dose thiazide should be considered to block or reduce urinary Ca loss,^(^ ^12^ ^)^ which may restore normal biochemical values, as well as limit the risk of development or progression of renal stones. In this circumstance, parathyroidectomy may not significantly reduce urinary Ca levels or renal stone risk, as the primary driver of parathyroid overactivity continues to be renal loss of Ca in urine.

Two studies have evaluated the calcium/phosphorus (Ca/P) ratio to help confirm or refine the diagnosis of PHPT. Both relied on the evaluation of serum phosphorus (P). In a single‐center, case–controlled, retrospective study including 97 patients with biochemically and histologically documented PHPT, Madeo and colleagues^(^ ^13^ ^)^ proposed a specific cut‐point for the albumin‐adjusted Ca/P ratio to easily identify subjects with PHPT without requiring measurement of PTH. A ratio cut‐point above 3.5, when Ca and P were measured in mg/dL, had a sensitivity of 89% and specificity of 91% for detecting patients in the cohort with either PHPT or NHPT. When the ratio was tested in 35 patients with NHPT, the sensitivity dropped to 67%, while retaining the same specificity as that of the entire cohort. The authors concluded that the Ca/P ratio could be of value for detecting PHPT in primary care settings or during high‐volume screening of patients as a result of its relative low cost because of its lack of need for measuring PTH. This tool might be less useful in NHPT because it is derived from single Ca and phosphate (PO4) measurements.

A more recent multicenter cross‐sectional study^(^ ^14^ ^)^ of 142 patients diagnosed with NHPT showed that the Ca/P ratio had a higher sensitivity (80.8%) than in the previous study, although the Ca/P ratio cut‐point was set at the lower level of 3.28. However, the positive predictive value was much lower at 47.4% compared to 73% in the previous study, suggesting a limited ability of this tool to identify PHPT when albumin‐adjusted Ca is within the normal range.

It is noteworthy that in both studies the Ca/P ratio had high negative predictive values of 88% and 95%, respectively, thereby indicating that the albumin‐adjusted Ca/P ratio might be a more reliable tool to exclude rather than confirm NHPT. The second study confirmed high accuracy of the Ca/P ratio in detecting PHPT when evaluating patients with both normocalcemic and hypercalcemic hyperparathyroidism together (sensitivity, 87.7%; specificity, 87.5%; positive predictive value, 82.4%; negative predictive value, 91.1%).

Guo and colleagues^(^ ^15^ ^)^ proposed another simple ratio called the PFindex to discriminate PHPT from vitamin D‐deficient secondary hyperparathyroidism. This index was defined as serum Ca × PTH/P, with Ca and P reported in mmol/L, and PTH in pg/mL. Guo and colleagues retrospectively assessed 128 surgically confirmed cases of PHPT, of whom 36 were normocalcemic. The study revealed that a PFindex of >34 was able to discriminate NHPT from vitamin D‐deficient secondary hyperparathyroidism with a sensitivity of 96.9% and a specificity of 97.6%.

All of these studies proposing various ratios to discriminate between NHPT, PHPT, and related disorders are limited by their retrospective design and lack of measurement of serum ionized Ca. This may have led to overestimation of NPHT cases in these studies. By not meeting the current definition of NHPT (as defined at the Fourth International Workshop on the Management of Asymptomatic Primary Hyperparathyroidism), many studies may have reported erroneous conclusions. The current definition of NHPT requires persistently normal serum total and ionized Ca over 3 to 6 months. Because PHPT is often characterized by fluctuating serum Ca levels in the upper‐normal and above‐normal ranges, a single Ca measurement in the upper‐normal range with high PTH should be interpreted as suspicious for PHPT rather than NHPT. Many studies have relied on single measurements of serum Ca and PTH, thus generating great confusion over the true prevalence and clinical impact of NHPT.

## Pathogenetic Hypotheses

**Fig 1:**
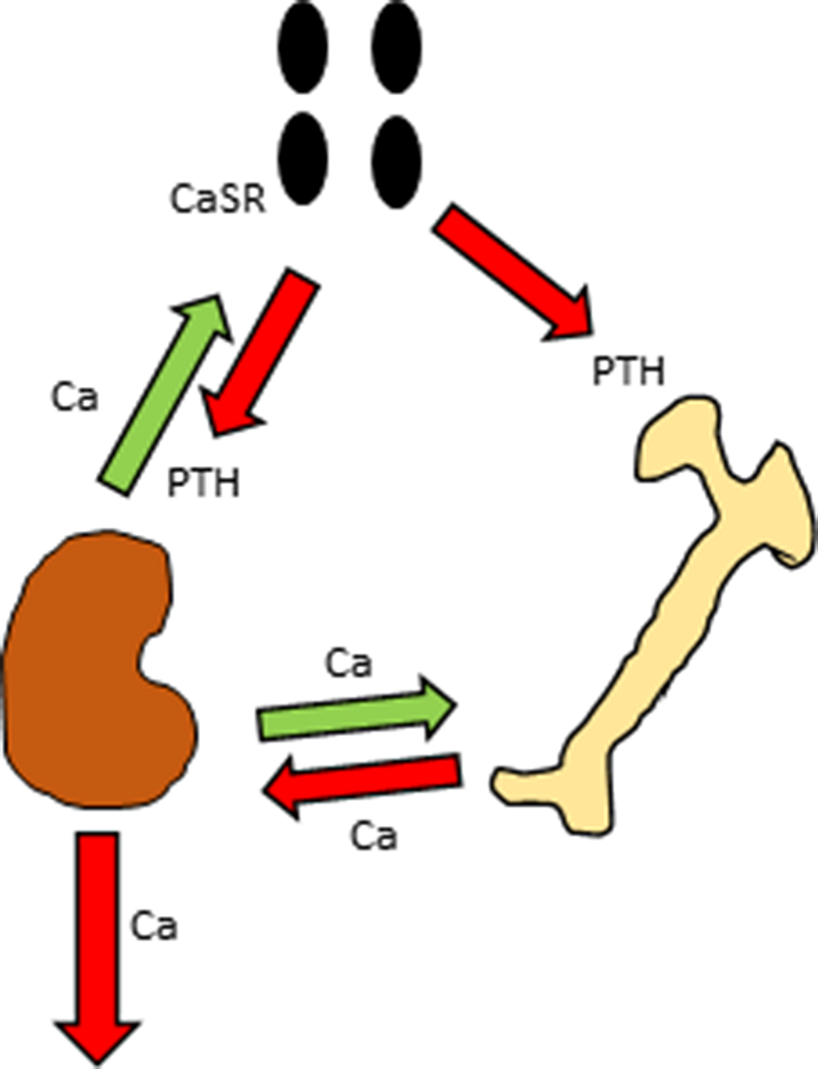
Normal physiology: Under normal physiology the parathyroid glands secrete PTH to stimulate bone turnover and reabsorb calcium (Ca) from the renal tubules. If serum Ca decreases, the parathyroid cell Ca‐sensing receptors (CaSRs) sense this, and induce the parathyroid glands to secrete more PTH to reabsorb more Ca from the kidneys and to mobilize more Ca from the bones to restore previous normal serum Ca. Given stable intestinal Ca absorption, the renal tubules reabsorb the amount of Ca needed to maintain negative feedback on the parathyroid glands, preserving normal PTH secretion and stable bone density. The parathyroid glands, small intestine, and kidneys work synergistically to maintain normal physiology.

The mechanism(s) underlying development of NHPT are currently not yet known. Several hypotheses have been proposed. These hypotheses are distinctly different from each other, so that it is difficult to establish a unifying conclusion on the pathogenesis of NHPT. Figure 1 shows normal Ca physiology and metabolism, and illustrates the homeostatic (dynamic) balance interplay between the parathyroid glands, bones, and kidneys. As shown, Ca reabsorption is maintained by the renal tubules to prevent oversecretion of PTH, and thereby preserve bone density and reduce the risk of hypercalciuria and kidney stones.

The most widely accepted view is that NHPT represents an early or milder form of classical PHPT.^(^ ^4^ ^)^ This hypothesis proposes that patients with NHPT gradually develop the classical form over time, with an eventual increase in serum and urine Ca levels. Given that the normal population has mean serum Ca concentrations ranging over an approximately 2 mg/dL interval, it is possible that increased serum PTH levels increase serum Ca to a mild extent within the normal range that is not clinically detectable within the general population, but significant enough to explain the pathophysiology of patients with NHPT. In other words, serum PTH levels would be inappropriately increased for the physiologic serum Ca level of the patient. This hypothesis presumes that those with serum Ca in the upper‐normal range might be more likely to convert to the hypercalcemic phenotype over time.^(^ ^16^ ^)^

A second view is that advancing age and menopausal status play a role in the increase in serum PTH levels. In 1994, Ledger and colleagues^(^ ^17^ ^)^ reported the phenomenon known as secondary hyperparathyroidism of aging. Secretory dynamics of PTH were evaluated in small groups of elderly postmenopausal (mean age 73 years) and younger premenopausal women (mean age 30). Neither group was taking hormone treatment or had medical conditions known to affect Ca metabolism. Serum 25(OH)D, 1,25‐dihydroxyvitamin D [1,25(OH)_2_D], and creatinine (creatinine clearance not reported) were similar in the two groups. Besides having greater mean baseline PTH values, elderly women showed a greater sensitivity of response of PTH levels to intravenous EDTA‐induced hypocalcemia. These abnormalities in the elderly women were completely reversed by a 1‐week treatment course with 1,25(OH)_2_D (calcitriol), which completely restored normal PTH dynamics by improving Ca absorption and transcriptionally suppressing PTH secretion. These PTH dynamics suggest that the more vigorous response of older postmenopausal women might be attributable to decreased suppression of baseline PTH secretion, which could also possibly be caused by mild parathyroid gland hyperplasia. Aging could lead to decreased 1,25(OH)_2_D action on intestinal Ca absorption or reduced 1,25(OH)_2_D levels because of compromised renal function, resulting in a chronic stimulus to the parathyroid glands. This study was more consistent with intestinal resistance to vitamin D action, which was promptly overridden by a short course of calcitriol.

Cosman and colleagues^(^ ^18^ ^)^ analyzed a cohort of postmenopausal women with osteoporosis to evaluate the regulation of PTH secretion by estrogen. Subjects receiving hormone therapy were found to have lower baseline PTH levels compared with those that were untreated. Moreover, the women receiving hormone therapy showed a blunted PTH response to the induction of EDTA‐mediated hypocalcemia. These findings suggested that parathyroid glands might be less sensitive to hypocalcemia under the influence of estrogen. Another study by Khosla and colleagues^(^ ^19^ ^)^ addressed whether the effect on PTH secretion was direct or indirect. EDTA‐stimulated PTH secretion was evaluated in 10 postmenopausal women before and after 3 days of estrogen therapy. The study showed that estrogens did not have significant effect on either basal or stimulated PTH secretion. The authors concluded that PTH levels were indirectly affected by estrogens, possibly by increasing intestinal and renal Ca absorption. PHPT and NHPT may be diagnosed more commonly in postmenopausal women based on the unmasking of mild hyperparathyroidism caused by loss of the protective effect of estrogen on bone, leading to a negative Ca balance. The mechanisms suggested in these two studies should be considered when diagnosing NHPT.

Invernizzi and colleagues^(^ ^20^ ^)^ showed that patients with NHPT maintained their PTH physiologic feedback in response to stimulation with PO4 or suppression with Ca. This study treated three patient cohorts with an oral peptone load rich in PO4 or an oral Ca load, respectively, in an effort to stimulate or suppress PTH secretion. The first cohort contained 22 patients with PHPT, the second cohort had 20 with NHPT, and the third cohort had 30 healthy controls without parathyroid dysfunction. In the first cohort with PHPT, oral Ca loading failed to decrease the mean serum PTH level, but in the second and third cohorts, serum PTH levels decreased significantly. In addition, the mean PTH response to PO4 loading was more pronounced in the PHPT cohort than in the NHPT or healthy control cohorts. Thus, it appeared that patients with NHPT may have preserved normal physiological feedback of minerals on PTH secretion. The results of this study were in contrast with several studies clearly supporting a partially preserved feedback of PTH secretion following Ca supplementation also in classical PHPT.^(^ ^21^ ^)^ Because PTH was only sampled during the oral Ca load (for 120 min), it is possible that the response to Ca in PHPT may take longer than what usually occurs in NHPT and healthy people. The rapid response to Ca in the NHPT cohort is consistent with physiology, pointing toward a form of secondary rather than PHPT.

Another hypothesis is that over‐production of PTH by patients with NHPT may be lower compared with that in patients with PHPT.^(^ ^4^ ^)^ Proving this hypothesis is difficult and, as yet, no substantial differences in basal PTH levels have been found between patients with PHPT and NHPT,^(^ ^22^ ^)^ except in one cohort studied by Maruani and colleagues.^(^ ^23^ ^)^ This study suggested that relatively lower secretion of PTH in NHPT might result in serum Ca concentrations remaining within the normal range. The study also proposed combined renal and bone resistance to PTH as a mechanism to explain NHPT, because, after matching the cohorts of PHPT and NHPT for PTH level, the normocalcemic cohort showed lower markers of bone turnover and lower serum 1,25(OH)_2_D levels, as well as lower capacity to increase urinary Ca reabsorption and blunted ability to increase urinary PO4 excretion.

The importance of vitamin D in NHPT has recently been highlighted by Wang and colleagues^(^ ^24^ ^)^ who identified 10 patients meeting criteria for NHPT out of 500 patients diagnosed with PHPT and another 400 patients screened for hyperparathyroidism after exclusion of obvious secondary causes of PTH elevation. They evaluated these 10 NHPT patients and 20 age‐, sex‐ and BMI‐matched control subjects, and reported that the NHPT patients had lower serum‐free 25(OH)D levels compared with the controls when measured by an immunometric assay. These patients and controls all had normal serum total 25(OH)D levels in the range of 30 to 40 ng/mL. PTH levels correlated with free, but not total 25(OH)D levels (*r* = −0.415; *p* < 0.05). The study concluded that some NHPT patients might have a form of secondary hyperparathyroidism caused by lower serum free 25(OH)D levels. This finding could be explained by higher concentrations of vitamin D‐binding protein that might mask vitamin D deficiency.

The function of the calcium‐sensing receptor (CaSR) might also play a role in NHPT. The CaSR polymorphism A986S is common in the general population and was demonstrated to have a significant effect on extracellular Ca level.^(^ ^25^ ^)^ Other single nucleotide polymorphisms have been associated to Ca levels, PTH elevation, and BMD.^(^ ^26^ ^)^ The genetic mechanisms behind these biochemical abnormalities resemble those of familial hypocalciuric hypercalcemia, with milder features in the general population. A recent study by Diaz‐Soto and colleagues^(^ ^27^ ^)^ compared the presence of specific polymorphisms in the CaSR in 41 patients with NHPT and 20 with asymptomatic PHPT. Participants were followed for 1 year and tested biochemically at least twice to ensure that they continued to have a stable diagnosis. The study demonstrated that the A986S polymorphism in the CaSR was an independent predictor of PTH level in NHPT, but not in asymptomatic PHPT. This polymorphism affects the intracellular domain of the CaSR, and appears to cause reduced CaSR function, thereby inducing lower sensitivity to extracellular serum Ca and stimulating increased PTH secretion in response. This resistance‐inducing polymorphism was not observed in the control group with asymptomatic PHPT, suggesting that these disorders might have different pathogenetic mechanisms.

**Fig 2:**
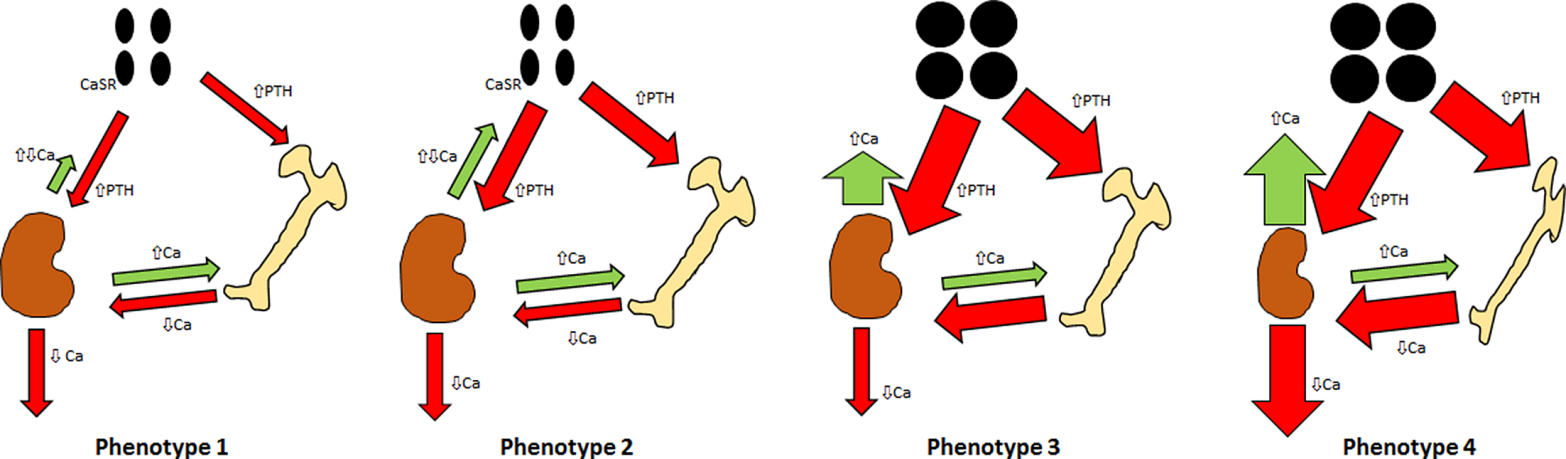
Patients with hyperparathyroidism appear to present with different phenotypes. It has not yet been observed that the recognized phenotypes progress to more severe phenotypes, or regress to less severe forms over time, with the possible exception of classical mild primary hyperparathyroidism regressing to normal when observed over years without surgery. Phenotype 1. Mild changes within the physiologic range: Subclinical increased renal calcium (Ca) losses stimulate the parathyroid glands to secrete increased PTH within the physiologic range. Phenotype 2. Normocalcemic hyperparathyroidism: PTH oversecretion stabilizes at a new set‐point, in which renal Ca filtration and reabsorption are increased to allow preservation of bone density, but not enough to cause Ca‐sensing receptor function to inhibit the parathyroid glands. Phenotype 3. Asymptomatic primary hyperparathyroidism: PTH oversecretion is associated with parathyroid gland enlargement and eventual parathyroid autonomy, with no evidence of bone or renal complications. Phenotype 4. Primary hyperparathyroidism: Clinical features of classical primary hyperparathyroidism with possible kidney malfunction and bone loss starting with the cortical sites.

Patients with hyperparathyroidism may present with different phenotypes. Figure 2 summarizes the various presentations of PHPT described in this section. The figure illustrates putative mechanisms sustaining PTH oversecretion based on renal tubular Ca loss, maintenance of NHPT, and possible further changes leading to PHPT (phenotypes 1 to 4). In phenotype 1, subclinical increased renal Ca losses stimulate the parathyroid glands to secrete increased PTH within the physiologic range. Bone is preserved based on PTH action on the kidneys, causing increased reabsorption of Ca from the proximal tubule. Kidney stones may be associated with increased renal Ca losses. This phenotype is mild enough that it may not be recognized clinically. In phenotype 2, PTH oversecretion stabilizes at a new set‐point, in which renal Ca filtration and reabsorption are increased sufficiently to allow preservation of bone density, but not enough to affect CaSR function to inhibit the parathyroid glands. Thiazides may help reduce the negative feedback on the CaSR. Kidney stones may be associated with increased renal Ca loss. In phenotype 3, PTH oversecretion is associated with parathyroid gland enlargement and eventual autonomy. A single adenoma may develop, or all four glands may become hyperplastic. Bone density is preserved because of sufficient renal Ca reabsorption to compensate for increased bone turnover. Increased Ca release from the skeleton and increased renal tubular reabsorption leads to mild hypercalcemia. Phenotype 4 portrays clinical features of classical PHPT, with hypercalcemia, hypercalciuria, increased bone turnover, and bone loss starting with the cortical sites. Given a sufficient and constant amount of dietary Ca, phenotypes 1 and 2 would explain why PTH remains increased, after having excluded all known secondary causes. Changes of normal aging might explain biochemical changes seen in some patients. It is not evident that NHPT evolves into PHPT or reverts to normal.

In summary, multiple pathogenetic hypotheses have been proposed, but none has been proven to explain some or all cases of NHPT (Table 1).

**Table 1:** Putative Mechanisms Underlying Normal Calcium and High PTH When Common Secondary Causes Have Been Ruled Out. Clinical and Biochemical Values of Each Study are Reported

Source	Hypothesis	Age (yrs)	F/M ratio	PTH (pg/ml)	sCa (mg/dL)	sP (mg/dL)	25(OH)D (ng/mL)	1,25(OH)_2_D (pg/mL)	Ionized calcium (mmol/L)	eGFR (ml/min)	Explanation	Implications
Lowe et al.^(^ ^22^ ^)^ (n = 7)^a^	Early form of PHPT	64 ± 2	95% F	96 ± 15	9.7 ± 0.2	3.4 ± 0.2	29 ± 3	62 ± 11	No	>40	PTH is secreted autonomously. It might progress to frank hypercalcemia.	Feedback on PTH is assumed not to be present.
Ledger et al.^(^ ^17^ ^)^ (n = 10)^b^	Aging	73.7 ± 0.6	100% F	3.8 ± 0.5 pmol/L	2.32 ± 0.02 mmol/L	1.22 ± 0.04 mmol/L	40.9 ± 4.8 nmol/L	65.8 ± 6.3 pmol/L	1.22 ± 0.04	Creatinine 83.1 ± 4.2 μmol/L	Aging may cause a reduced effect of 1,25‐OH vitamin D on intestinal calcium absorption, thus raising PTH inducing a chronic stimulus on the parathyroid glands	Feedback on PTH might be restored with 1,25‐OH vitamin D, through a greater calcium absorption
Cosman et al.^(^ ^18^ ^)^ (n = 9)^c^	Low estrogen status (menopause)	61 ± 3	100% F	5.08 ± 0.51 pmol/L	NR	NR	58 ± 6 nmol/L	77 ± 8 pmol/L	1.281 ± 0.022	NR	Low estrogens have been associated with a lower sensitivity to hypocalcemia. Women receiving HT were found to have lower PTH in the setting of induced hypocalcemia. Besides the known protective effect on bone, better intestinal and renal calcium absorption, rather than direct effects on parathyroid glands seems to link estrogen with PTH dynamics.	Feedback on PTH is dependent on estrogen status
Vincent et al.^(^ ^19^ ^)^ (n = 10)		76.4 ± 1.9	100% F	≈5‐7.5 pmol/L	2.36 ± 0.02 mmol/L	NR	NR	NR	≈1.2‐1.25 mmol/L	Creatinine 88.4 ± 3.2 μmol/L		
Invernizzi et al.^(^ ^20^ ^)^ (n = 20)	Nutritional (feedback preserved)	55.3 ± 6.2	65% F	≈100	NR	≈3	36.7 ± 6.78	NR	≈1.3	81 ± 7.1	Oral calcium loading reduced PTH rapidly as opposed to PHPT, where this did not occur over 120 min. Oral phosphate loading increased PTH similarly in NHPT and controls.	That would point toward an unrecognized form of secondary hyperparathyroidism, with a physiologic response to minerals
Maruani et al.^(^ ^23^ ^)^ (n = 34)	A partial resistance to PTH from bone and kidney in NHPT but not in PHPT	53 ± 14	68% F	75 ± 19	9.62 ± 0.22	3.3 ± 0.4	33(17‐86)	103 ± 30 pmol/L	1.32 ± 0.03	Creatinine 0.80 ± 0.13 mg/dL	Lower secretion of PTH so that calcium can remain in the normal range	Lower impact on biochemistry and bone turnover markers
Wang et al.^(^ ^24^ ^)^ (n = 10 NHPT *vs* n = 20 controls)^e^	Low free 25‐hydroxivitamin D	59.9 ± 5.4	90% F	98.1 ± 31.7	9.3 ± 0.3^d^	NR	31.9 ± 1.7 vs 32.7 ± 3.3 *(total Vit.D)* 5.0 ± 0.9 vs 6.2 ± 1.3 *(free Vit. D)*	NR	NR	‘normal’	PTH levels correlate with free, but not total vitamin D	It is an unrecognized secondary form, therefore feedback on PTH is preserved.
Diaz‐Soto et al.^(^ ^27^ ^)^ (n = 41, 17 of whom with A986S polymorphism of CaSR)	Altered parathyroid sensing	63 ± 11	83% F	103 ± 25	9.23 ± 0.43	3.39 ± 0.48	34.0 ± 10.9	47.2 ± 15.8	1.22 ± 0.04	89.3 ± 28.1	Some polymorphisms in CaSR may lower sensitivity of the CaSR to serum calcium levels, thereby physiologically leading to a greater secretion of PTH.	No feedback on PTH.

To convert calcium from milligrams per deciliter to millimoles per liter, multiply by 0.25.

To convert phosphorus from milligrams per deciliter to millimoles per liter, multiply by 0.323.

To convert 25‐hydroxyvitamin D from nanograms per milliliter to nanomoles per liter, multiply by 2.496.

To convert 1,25‐dihydroxyvitamin D from picograms per milliliter to picomoles per liter, multiply by 2.6.

To convert PTH from picograms per milliliter to picomoles per liter, multiply by 0.105.

To convert creatinine from μmol per liter to millimgrams per deciliter, multiply by 0.113.

^a^ Characteristics of patients who became hypercalcemic.

^b^ Values of the ‘elderly women group’ who responded to calcitriol are reported.

^c^ Basal values of untreated postmenopausal women undergoing EDTA‐induced hypocalcemia.

^d^ Albumin‐adjusted calcium.

^e^ NHPT data are shown.

CaSR = Calcium‐sensing receptor; NHPT = normocalcemic primary hyperparathyroidism; HT = hormone therapy; NR = not recorded.

## Prevalence

Population‐based studies have generally suggested that NHPT is a rare condition. Estimates of prevalence have been variable, presumably because of different selection criteria used to identify NHPT patients^(^ ^28^, ^29^, ^30^, ^31^, ^32^, ^33^, ^34^, ^35^ ^)^ (Table 2). The prevalence of the condition in these population‐based studies has been estimated to range between 0.1% and 6%. Some studies show that the prevalence of NHPT may vary over time, suggesting that NHPT might be reversible, or that the initial diagnosis was incorrect. In the largest population‐based study with a follow‐up over 8 years,^(^ ^32^ ^)^ NHPT was detected in 3.1% of subjects at baseline. After 8 years, more than 75% of those classified initially as NHPT either returned to normal or were diagnosed with secondary hyperparathyroidism.

**Table 2:** Population‐Based Studies of the Prevalence and Complications of Normocalcemic Hyperparathyroidism

Study	Years	Country	Total cohort (*n*)	Baseline prevalence	Follow‐up duration (years)	Follow‐up prevalence	Mean Age	F/M ratio	PTH (pg/mL)	S‐Ca (mg/dL)	S‐P (mg/dL)	S‐25‐(OH)D (ng/mL)	S‐1,25‐(OH)_2_D (pg/mL)	eGFR (mL/min)	Bone complications	Renal stones	Notes
Lundgren et al.^(^ ^28^ ^)^	1991‐1992	Sweden	5771	0.54%	NR	NR	Range 55‐75 (overall population)	100% F	85.1 ± 40.64	2.52 ± 0.07 mmol/L	NR	NR	NR	NR	NR	NR	Used both ionized calcium and albumin‐adjusted calcium. Tested on 3 or more occasions.
Berger et al.^(^ ^29^ ^)^	1995‐2007	Canada	1872	3.31%	NR	NR	71.7 ± 2.4 (69.3‐74.2)	NR	>10.2 pmol/L, no mean values provided	Normal	NR	70 ± 25nmo/L (all participants)	NR	79.2 (76.3‐82.2)	No difference from control group; elevation of BSAP similar to secondary hyperparathyroidism; total hip BMD lower in those with higher PTH levels	12.4% of males in total cohort, 6.3% of females in total cohort; unknown in NHPT group	6.45% taking glucocorticoids, 45% on antiresorptive drugs, 26% on diuretics. Urine calcium/urine creatinine=0.42. Tested only on one occasion.
Kontogeorgos et al.^(^ ^30^ ^)^	1995‐2009	Sweden	608	2%	13	0.2%	53.3 ± 9.0	NR	73.4 ± 14.2^a^	2.34 ± 0.08 mmol/L^a^	NR	≥20 65.8 ± 13.8 nmol/L^a^	118.5 ± 42.7 pmol/L^a^	Normal	No difference in past history of fracture; 2/7 sustained fractures during follow‐up	NR	Calcium not adjusted for albumin; 1 patient developed hypercalcemia with PTH inappropriately normal and increased vitamin D. No use of ionized or urinary calcium. Single measurements of blood samples.
Palermo (OPUS Study) et al.^(^ ^31^ ^)^	1999‐2001	5 European Centers	2419	0.1% (1 patient)	6	0	NR	100% F	NR	NR	NR	≥20	NR	>60	NR	NR	Albumin‐adjusted calcium only. Tested only once at baseline.
Cusano DHS et al.^(^ ^32^ ^)^	2000‐2010	US	3450	3.1%	8	0.6%	41.3 ± 12	38% F	>55 94.8 ± 46	9.3 ± 0.6	3.1 ± 0.6	≥20 30.1 ± 11	NR	>60 (creatinine 0.93 ± 0.1)	No difference in OPG or CTX between patients and controls	NR	Albumin‐adjusted calcium only; no thiazides or lithium; single lab value for basal cohort. Of 64 patients with follow‐up, only 1 developed hypercalcemia (1.5%), and 49 (76.5%) were reclassified as normal or with SHPT. Lab measurements only once at baseline and follow‐up.
Cusano MrOS et al.^(^ ^32^ ^)^	2000‐2002	US	2503	0.36%	NR	NR	70 ± 6	100% M	>66 77.5 ± 13	9.4 ± 0.6	3.0 ± 0.5	≥20 25.2 ± 5	NR	>60 1.0 ± 0.1	No differences from normal population re: BMD, P1NP, CTx or TRAP‐5b	NR	Albumin‐adjusted calcium only; no thiazides; single basal lab value.
Rosario et al.^(^ ^33^ ^)^	2009‐2014	Brazil	676	0.6%	NR	NR	53	80% F	95.3(76‐112)	9.8	NR	≥30	NR	>60	Exclusion criteria: History of pathological fractures	Exclusion criteria: History of nephrolithiasis or nephrocalcinosis	Use of ionized calcium; 80% (4/5 patients) NHPT showed evidence of pathologically confirmed parathyroid adenoma(s) at thyroid‐related surgery. Lab measurements repeated a second time, but temporal distance unknown.
Vignali et al.^(^ ^34^ ^)^	2010	Italy	685	0.4%	NR	NR	47.0 ± 22.9	100% M	133 ± 5	8.9 ± 0.1	NR	≥30 37.5 ± 5.3	NR	>60 96.5 ± 26.1	NR	NR	Albumin‐adjusted calcium only; Exclusion of bisphosphonates and thiazides. 1 out of the 3 patients with NHPT had an estimated calcium intake of 107 mg/day.
Garcia‐Martin et al.^(^ ^35^ ^)^	Unknown (1 year)	Spain	100	6%	1	6%	56.3 ± 3.2	100% F healthy, PMP^2^	81.3 ± 10	8.9 ± 0.2	3.3 ± 0.4	≥30	NR	80 ± 13	No difference from control group; BMD estimated by QUS; NHPT and SHPT cohorts showed negative correlation of PTH and BMD by QUS	0% at baseline and follow‐up	Albumin‐adjusted calcium; single lab value for definition; no other criteria specified other than healthy.

BSAP = Bone‐specific alkaline phosphate; CTX = C‐terminal telopeptide; NR = not recorded; OPG = Osteoprotegerin; P1NP = procollagen 1 N‐terminal propeptide; PMP = postmenopausal; QUS = quantitative ultrasound; SHPT = secondary hyperparathyroidism; TRAP‐5b = tartrate‐resistant acid phosphatase 5b.

^a^ Biochemical data from patients at follow‐up.

Analysis of the characteristics of the participants included in all available studies shows that there is a higher prevalence of NHPT reported in smaller cohorts, and that most of these studies have not rigorously excluded all known causes of secondary hyperparathyroidism. Table 2 also shows that vitamin D thresholds were different in each study and ionized Ca was not measured consistently. Also, observed discrepancies among the different cohorts may be based in part to nutritional status, which may vary with age, sex, and country of origin.

Unexpected findings were reported in the Osteoporosis and Ultrasound Study (OPUS).^(^ ^31^ ^)^ Among the 2419 women included in this study, only one case of NHPT was identified. This patient was retested later, and found to no longer meet criteria for NHPT. The study reported that the prevalence of NHPT was zero in this fairly large cohort. However, this is not the only study showing a higher prevalence of NHPT at baseline and a significantly lower prevalence during follow‐up over several years (Table 2). These studies suggest that repeating laboratory evaluation over time is essential in confirming the diagnosis of NHPT.

## Classical Complications

In contrast with what has been observed in population‐based studies, both the number of cases of NHPT and reported complication rates are greater in series from referral centers for metabolic bone disease, thus implicitly suggesting a significant selection bias. Table 2 highlights the prevalence of complications associated with NHPT in population‐based studies; Table 3 shows bone and renal complications reported in referral center cohorts. Notably, population‐based studies to date have shown either no evidence or less evidence of substantial bone or renal complications. The pattern of changes in bone metabolism identified in population‐based NHPT cohorts was similar to those seen in normal subjects or patients affected with secondary hyperparathyroidism. There is no evidence to suggest increased risk of kidney stones in the population‐based studies. Thus, it appears that NHPT identified solely by biochemical criteria, rather than by recognized bone disorders or renal stones, may not be associated with significant complications. It should be noted, however, that both population‐based studies and referral center cohorts used variable cut‐offs for 25(OH)D sufficiency without consistently measuring ionized Ca. Because these two parameters are crucial in the definition of NHPT, it is easy to misclassify subjects as having NHPT that otherwise would be diagnosed with PHPT; this results in incorrect estimates of the prevalence and complications of the disease.

**Table 3:** Referral‐Center Based Studies of the Prevalence and Complications of Normocalcemic Hyperparathyroidism

Study	Years	Country	Total cohort (*n*)	Prevalence	Mean Age	F/M ratio	S‐Ca (mg/dL)	S‐P (mg/dL)	S‐25‐OH Vit D (ng/mL)	eGFR (mL/min)	Bone Complications	Renal Stones	Notes
Amaral et al.^(^ ^41^ ^)^	Unknown	Brazil	33	NR	64 ± 14	79% F	9.58 ± 0.44	NR	≥30	>60	Osteoporosis: unknown. 15% had fractures	18%	LS and FN BMD comparable between NHPT and PHPT. 1/3 distal radius BMD lower in PHPT vs NHPT. Similar prevalence of nephrolithiasis in NHPT and PHPT. Single determination of lab values.
Maruani et al.^(^ ^23^ ^)^	1990‐1998 Prospective	France	34	5.2%	53 ± 14	68% F	9.62 ± 0.22	3.3 ± 0.4	33 (17–86)	Normal mean creatinine (0.80 ± 0.13 mg/dL)	Radiographic bone demineralization (18%)	35%	NHPT: hypophosphatemia (9%), lower markers of bone turnover and 1,25 dihydroxyvitamin D compared with PHPT. Single assessment of ionized calcium.
Tordjman et al.^(^ ^44^ ^)^	1998‐2003 Retrospective	Israel	32	NR	61 ± 11	84% F	9.8 ± 0.46	2.9 ± 0.47	22.7 ± 7.8	99 ± 22	38% osteoporosis	19% had elevated UCa >300 mg/d. 9% nephrolithiasis.	CrCl calculated from a 24‐h urine collection. 20 patients not undergoing surgery had stable serum calcium, mean follow‐up of 4.1 years (range 1–13 years, not stated how frequently).
Silverberg et al.^(^ ^37^ ^)^	2003 Retrospective	US	22	NR	57 ± 10	91% F	9.60 ± 0.08	3.32 ± 0.50	34 ± 3	NR	45% osteoporosis; 5% fragility fractures	14%	eGFR not shown, but normal renal function (mean creatinine 0.9). Single determination of lab values
Lowe et al.^(^ ^22^ ^)^	1998‐2005 Prospective	US	37	NR	58 ± 12	95% F	9.6 ± 0.1	3.3 ± 0.1	33 ± 1	>40	57% osteoporosis; 11% fragility fractures	14%	Patients followed for a mean of 3.1 years, 19% became hypercalcemic. Patients were evaluated yearly, but the initial classification in NHPT based on a single measurement.
Wade et al.^(^ ^40^ ^)^	1999‐2008 Retrospective	US	8	NR	60 (38‐68)	63% F	9.8	NR	21 (9.4–54.0)	NR	25% osteoporosis; 13% fractures	25%	Classified as having NHPT if they had no elevated total serum calcium values during the 3 months prior to surgery. 86% of patients with apparent NHPT had elevated ionized calcium.
Koumakis et al.^(^ ^45^ ^)^	2008‐2010 Prospective	France	39	NR	66.1 ± 9.1	92% F	10.06 ± 0.32	2.88 ± 0.50	34.3 ± 7.2	80.7 ± 17.9	92% osteoporosis; 40% fractures	18%	59% of NHPT had elevated ionized calcium. All patients underwent a thiazide diuretic test and oral or iv calcium load test to confirm autonomous PTH secretion.
Marques et al.^(^ ^38^ ^)^	2011 Retrospective	Brazil	14	7.8% of women coming for osteoporosis screening	61 ± 15	100% F	9.4 ± 0.4	NR	42 ± 10	>40	36% osteoporosis; 21% fractures	29%	BMD and fractures not different from controls. Kidney stones occurred more frequently in NHPT (29% vs 0.7%). Diagnosis of NHPT was based on at least 2 samples of corrected total calcium.
Cakir et al.^(^ ^39^ ^)^	2011 Prospective	Turkey	18	NR	49.9 ± 2.4	89% F	9.7 ± 0.1	3.1 ± 0.1	≥20	NR	47% osteoporosis; fracture: unknown	11%	Primary end point was insulin resistance in patients with NHPT. Single determination of lab values.
Siprova et al.^(^ ^43^ ^)^	2008‐2014 Retrospective	Czech Republic	137	NR	61 (26‐85)	81% F	Normal, <10.4 mg/dL	Range (1.52‐4.61)	≥20	‘Renal insufficiency’ as exclusion criteria	42% had reduced bone density (unspecified if osteoporosis or osteopenia)	4%	36 NHPT (26.2%) converted to PHPT within 6 years. No correlations found among PTH, S‐Ca, and S‐P. Single determination of lab values.
Palermo et al.^(^ ^47^ ^)^	2016‐2018 Prospective	Italy	41	NR	63.8 ± 9.3	91% F	9.4 ± 0.4	3.2 ± 0.5	36.7 ± 6.6	83 ± 23	BMD similar to controls at all sites, % of osteoporotic patients unknown. Overall fracture risk not increased: RR 1.12 (0.56 – 2.27).	13%	NHPT subjects were age‐ and sex‐matched with PHPT and controls. S‐P levels lower in NHPT than controls. Lower distal radius BMD in PHPT compared to NHPT and controls. Fracture risk increased in PHPT. NHPT diagnosed with at least 2 different biochemical determinations, at least 3 months apart).

CrCl = Creatinine Clearance; FN = femoral neck; LS = lumbar spine; NHPT = normocalcemic primary hyperparathyroidism; NR = not recorded; PHPT = primary hyperparathyroidism; RR = relative risk; S‐Ca = serum calcium; S‐P = serum phosphorus.

Patients with NHPT having low bone density, osteoporosis, or low‐trauma fractures have mostly been reported in small cohorts in selected referral centers that evaluated limited numbers of cases of NHPT. Most of these studies ranged between 6 and 37 patients. These data were summarized and discussed in the review by Cusano and colleagues,^(^ ^4^ ^)^ showing that the prevalence of osteoporosis in NHPT was variable, ranging from as low as 15% to as high as 57%. Fragility fractures appeared to have similarly variable rates, occurring in 11% to 21% of subjects diagnosed with NHPT. The study by Siprova and colleagues^(^ ^43^ ^)^ reported the largest number of NHPT patients with low bone density, osteoporosis, or fractures. This study included 137 NHPT patients (81% female), of whom 36 converted to PHPT within 6 years. Of these, 42% were described as having reduced bone density, without specifying whether they had osteopenia or osteoporosis. Bone quality may also have been affected in these patients, with increased risk of fracture.

Charopoulous and colleagues^(^ ^36^ ^)^ evaluated the effects of NHPT and PHPT on cortical and trabecular sites in postmenopausal women using pQCT scans of the tibia in postmenopausal women. They demonstrated catabolic actions on both cortical and trabecular compartments in NHPT and PHPT, as well as deterioration in cortical geometry in both phenotypes, with relative preservation of trabecular architecture. These findings suggest that bone loss occurs silently over time in NHPT. As a result, NHPT may be responsible for the onset and progression of the same skeletal complications as described in classical PHPT.

Less is known regarding the prevalence and causes of nephrolithiasis in NHPT. In referral cohorts, rates of renal stones are reported to be as high as 18% to 29%.^(^ ^41^, ^42^, ^43^, ^44^ ^)^ The prevalence of renal stones in NHPT in most studies is 10% or slightly more (Table 3), which is not much different from current estimates in the US population (10.1%) based on the National Health and Nutrition Examination Survey database.^(^ ^46^ ^)^ In some cohorts, the small numbers of patients and the heterogeneous ways of defining or excluding hypercalciuria in these cohorts may have led to overestimation of the prevalence of nephrolithiasis. The study by Siprova and colleagues^(^ ^43^ ^)^ suggested that nephrolithiasis was more common in patients with hypercalcemic PHPT, with 22% reported to have kidney stones, whereas only 4% of the NHPT patients had nephrolithiasis.

It is still not yet clear whether NHPT progresses toward hypercalcemic PHPT in some or all patients with the development of persistent hypercalcemia over time. The study by Schini and colleagues^(^ ^42^ ^)^ summarized the literature available on the natural history of biochemical changes in NHPT. This review demonstrated highly variable rates of evolution of NHPT into classical PHPT ranging between zero and 19%, so that it was difficult to draw a definitive conclusion. The reasons for such great variability may include incorrect diagnosis at baseline based on single biochemical measurements or selection bias. Schini and colleagues^(^ ^42^ ^)^ suggested that strict application of the international diagnostic criteria for NHPT would have resulted in a prevalence of zero in their cohort, despite a large population of 6280 subjects. They demonstrated that patients classified as having NHPT had frequent oscillation of their serum Ca values during the follow‐up period, sometimes going above the upper reference limit. This, by definition, would exclude the diagnosis of NHPT and classify those subjects as having PHPT. As a consequence, they suggested revising the criteria for diagnosing NHPT because the persistence of normal serum Ca and ionized Ca levels over time may not truly represent this disease phenotype. Instead, they proposed identifying these patients by using normal mean serum Ca levels. None of their 11 subjects with NHPT by this definition developed persistent hypercalcemia, but a pattern of intermittent hypercalcemia was seen in seven of them. All patients with PHPT have fluctuation in their serum Ca levels and occasionally would be expected to have levels within the normal range; therefore, it may be incorrect to identify this pattern as that of NHPT. This, instead, is more likely consistent with a very mild form of PHPT. Also, the main limitations behind these observations are that ionized Ca was not measured, and that blood samples were collected randomly without fasting, thus possibly resulting in an incorrect estimation of NHPT cases.

Palermo and colleagues performed a cross‐sectional study to characterize the clinical, biochemical, and radiological profile of NHPT.^(^ ^47^ ^)^ They recruited and analyzed three consecutive groups: 41 patients with PHPT, 47 patients with NHPT, and 39 age‐ and sex‐matched controls. The NHPT cohort was precisely defined compared with other studies, meeting the criteria of the Fourth International Workshop. The authors found progressively lower PO4 concentrations in the control, NHPT, and PHPT groups, respectively. Serum ionized Ca was lower in NHPT than in control subjects, despite greater values of serum 25(OH)D. PTH levels and bone turnover markers did not differ between PHPT and NHPT. NHPT patients had similar BMD at the lumbar spine, hip, and one‐third distal radius, and similar prevalence of morphometric vertebral fractures (28% versus 23%; NS) and nephrolithiasis (13% versus 3%; NS) compared with control subjects. The PHPT patients had a greater number of morphometric vertebral fractures (60%) and lower BMD at the one‐third distal radius compared with controls. Of note, 79% of NHPT patients met the criteria for asymptomatic hyperparathyroidism. The study concluded that the biochemical findings were consistent with an intermediate phenotype between PHPT and the normal population. This study was unable to identify hyperparathyroidism‐related bone disease in the cohort with NHPT. A longer follow‐up study of these three cohorts would be useful in exploring the natural history of complications in NHPT, especially in clarifying whether newly diagnosed NHPT represents a very early biochemical manifestation of PHPT.

## Nonclassical Manifestations

Recently, interest has developed regarding nonclassical manifestations of NHPT, but data are very limited in this regard. A recent study from China^(^ ^48^ ^)^ showed that NHPT may be associated with a greater risk of hypertension. The authors suggested a possible role for PTH in increasing blood pressure by acting directly on vessels or through the renin–angiotensin–aldosterone system, and concluded that surgery with normalization of PTH levels might be beneficial in controlling hypertension. Recent evidence has shown that the PTH1 receptor is found on human adrenocortical cells, and the mineralocorticoid receptor on the parathyroid glands.^(^ ^49^ ^)^ NHPT does not appear to be associated with ischemic heart disease, as one study excluded an influence of NHPT on the coronary calcium score, which has been correlated with coronary artery disease.^(^ ^50^ ^)^ Another recent study showed no improvement in cerebrovascular or neuropsychological function in patients with NHPT after parathyroidectomy.^(^ ^51^ ^)^

One study has examined the relationship between NHPT and quality of life.^(^ ^52^ ^)^ This study addressed both physical and mental aspects of patients with NHPT and PHPT. In NHPT, parathyroidectomy led to mild improvement in certain physical symptoms (Role‐Physical scale, SF‐36‐v2 instrument), but no effect was seen on mood‐related symptoms. Nonspecific symptoms such as thirst and fatigue also improved in patients with NHPT who underwent parathyroidectomy. Conversely, in the PHPT cohort, parathyroidectomy improved mental status, in addition to ameliorating physical symptoms and improving a greater number of nonspecific symptoms (anxiety, fatigue, muscle‐wasting, thirst, weight loss, loss of appetite, bone pain, constipation, and headaches) compared with the NHPT cohort. However, because a healthy control group was not included in the study, definitive conclusions could not be drawn regarding the outcomes observed in the NHPT cohort.

## Surgical and Medical Outcomes

Few data about the effects of parathyroidectomy or medical management on NHPT have been published to date. In the largest study, Pandian and colleagues^(^ ^53^ ^)^ confirmed that NHPT is characterized by a higher prevalence of multigland disease, which has been associated with lower cure rates.^(^ ^54^ ^)^ Smaller gland size may also be more common in NHPT as compared with PHPT.^(^ ^55^ ^)^ These findings have implications for the preoperative and surgical localization of the pathological glands. To address this issue, Pandian and colleagues^(^ ^53^ ^)^ concluded that routine bilateral neck exploration was necessary, in addition to using intraoperative PTH monitoring (ioPTH) in all patients affected by NHPT. Alternatively, another study suggested^(^ ^55^ ^)^ proceeding with bilateral neck exploration (BNE) if ioPTH did not drop by 50% within 10 min of removal of the suspected overactive parathyroid gland(s). However, this approach could be overly aggressive in the setting of NHPT. The authors^(^ ^55^ ^)^ suggested that surgeons should have a low threshold for BNE in NHPT because their case series of 119 NHPT patients required a higher conversion from a targeted approach to BNE compared with the conversion in their patients with PHPT (13% versus 4%). The authors acknowledged that long‐term studies are needed to clarify the role of surgery in NHPT. The basis for recommending ioPTH monitoring with or without BNE is the observation that the decline in ioPTH observed in patients with NHPT may be slower than in patients with PHPT. This slower decline has been associated with lower cure rates.^(^ ^56^ ^)^

Regarding improvement of bone complications after parathyroidectomy, one study^(^ ^57^ ^)^ reported that increased PTH levels were common (46.5%) after parathyroidectomy for NHPT, and that they were associated with a lack of BMD improvement after parathyroid removal. Postoperative hyperparathyroidism is not an uncommon finding after parathyroidectomy for classical PHPT, with a mean prevalence of 23.5% among various studies, mostly related to vitamin D deficiency or insufficiency or lack of Ca through diet or supplements.^(^ ^58^ ^)^ As opposed to PHPT where this finding invariably represents a form of secondary hyperparathyroidism because the Ca normalizes, elevated PTH levels found after surgery for NHPT may be the only sign of disease persistence, possibly hinting at a previously unidentified cause of secondary hyperparathyroidism involving multiple glands. Another study showed a mild (2.3% ± 5.0%), but significant increase in lumbar spine BMD in NHPT patients undergoing successful parathyroidectomy.^(^ ^45^ ^)^ A large surgical single‐center retrospective study found that after parathyroidectomy, as many as 41.7% and 40.0% of patients showed improvements in BMD and kidney stones, respectively.^(^ ^59^ ^)^ However, this study did not provide *T*‐scores or BMD percentage changes.

In contrast, two studies have been published regarding pharmacologic treatment of bone and renal complications associated with NHPT. One study evaluated the effects of oral alendronate and cholecalciferol in a small cohort of postmenopausal women.^(^ ^60^ ^)^ All skeletal sites improved with alendronate therapy after 12 months, with lumbar spine BMD improving by 4.7%, total hip BMD improving by 4.0%, and femoral neck BMD improving by 2.6%. The control group receiving cholecalciferol alone experienced significant BMD loss of 1.6% at the lumbar spine, 1.4% at the total hip, and 1.7% at the femoral neck. The other study investigated the effectiveness of cinacalcet in improving nephrolithiasis.^(^ ^61^ ^)^ Although the study consisted of only 10 patients with hyperparathyroidism, 6 of whom were normocalcemic, cinacalcet reduced the number and size of urinary stones in both the hypercalcemic and normocalcemic groups over a follow‐up period of 10 months.

A single case of parathyroid carcinoma was reported to present with NHPT.^(^ ^62^ ^)^ A large surgical series^(^ ^53^ ^)^ compared outcomes in NHPT (*n* = 733) versus PHPT (*n* = 6836) patients. Among those with parathyroid cancer or atypical adenoma (*n* = 212), 26.8% (*n* = 57) were categorized as having NHPT. This histologic category was not further analyzed to distinguish between cancers and atypical adenomas. The authors commented that this unexpected finding might have been caused by pathological misclassification.

## Summary

There is a strong scientific and clinical need to determine a precise definition for NHPT. Ca, PTH, vitamin D ranges, P, and the number of sequential Ca measurements over a specified period need to be derived by consensus. Short of such an endeavor, there will always be misclassification of NHPT and PHPT. This is a major obstacle to the ability to phenotype this condition, understand its pathophysiology and complications, and make clinical management recommendations. The pathophysiology of NHPT is heterogeneous. A registry of NHPT patients may help sort out differences in pathophysiology in various subgroups of patients.

Lack of rigorous diagnostic criteria has led to reporting of heterogeneous rates of bone and renal complications. This could be one of the reasons why NHPT may present asymptomatically or with traditional complications as described in PHPT. The symptomatic form of NHPT has mainly been reported by referral centers for metabolic bone disorders; therefore, selection biases could have led to an overestimation of the clinical impact of the disease.

NHPT is a biochemical diagnosis of exclusion, and laboratory testing over time is necessary to distinguish this from secondary hyperparathyroidism. Follow‐up frequently reveals resolution of hyperparathyroidism.

The natural history of NHPT remains unknown. Population‐based studies have shown that a large proportion of patients suffer from secondary rather than PHPT because the disease resolves with time and nonpharmacological interventions. Longitudinal follow‐up is needed to confirm the diagnosis and evaluate proper therapeutic options. Caution should be used in making the diagnosis and in recommending early surgical intervention, unless clearly indicated. Rigorous use of diagnostic criteria will allow a better assessment of features of the disorder and its natural history.

## Conclusions

- A precise definition for NHPT has been derived by consensus. Prospective studies are needed that include patients based on this definition.
- The pathophysiology of NHPT may be heterogeneous.
- NHPT is a biochemical diagnosis of exclusion, with appropriate long‐term follow‐up frequently revealing resolution of hyperparathyroidism.
- The natural history of NHPT remains unknown.
- Caution should be used in recommending surgery for NHPT, but when surgery is done, multigland disease has been reported.

### Areas for Future Research

Prospective studies with stringent diagnostic criteria as recommended by the Fourth International Workshop on the Management of Asymptomatic Primary Hyperparathyroidism (ie, repeated measurements of albumin‐corrected and ionized Ca over 3 to 6 months) will be necessary to elucidate the real impact of NHPT on the onset and progression of renal, bone, and nontraditional manifestations of this disorder. New biomarkers may help to simplify the diagnostic process and differentiate NHPT from PHPT without requiring 3 to 6 months of follow‐up. The role of serum PO4 or bone turnover markers could be further investigated to derive accurate diagnostic tools.

Recent studies suggest that skeletal complications of NHPT may be less common than previously reported, and that parathyroidectomy may lead to small improvements of BMD. Antiresorptive treatment with bisphosphonates or denosumab might produce similar or greater BMD gains. This evidence suggests that patients with NHPT associated with osteoporosis might benefit more from pharmacologic therapy than surgical treatment.

## Author contributions

**Guido Zavatta:** Conceptualization; data curation; formal analysis; investigation; methodology; project administration; resources; software; writing‐original draft; writing‐review and editing. **Bart Clarke:** Conceptualization; data curation; formal analysis; investigation; methodology; project administration; resources; software; supervision; writing‐review and editing.

## Peer Review

The peer review history for this article is available at https://publons.com/publon/10.1002/jbm4.10391.
